# 1367. Resistance to Ceftriaxone and Ciprofloxacin in Nontyphoidal *Salmonella* from Humans and Relatedness to Isolates from Animal Sources

**DOI:** 10.1093/ofid/ofac492.1196

**Published:** 2022-12-15

**Authors:** Nkuchia M M’ikanatha, Sameera Sayeed, Molly M Leeper, Jason P Folster, Sameh Boktor, Louise F Watkins, Yezhi Fu, Nagaraja Thirumalapur, Lisa Dettinger, Nicole M Hackman, Heather Tate, Edward G Dudley

**Affiliations:** Pennsylvania Department of Health, Harrisburg, Pennsylvania; Pennsylvania Department of Health, Harrisburg, Pennsylvania; CDC/NCEZID/DFWED/EDLB/PulseNet, Atlanta, Georgia; Centers for Disease Control and Prevention OID/NCEZID/DFWED/EDLB, Atlanta, Pennsylvania; Pennsylvania Department of Health, Harrisburg, Pennsylvania; Centers for Disease Control and Prevention, Atlanta, Georgia; Penn State College of Agricultural Sciences, University Park, Pennsylvania; Pennsylvania Department of Agriculture, Harrisburg, Pennsylvania; Pennsylvania Department of Health, Harrisburg, Pennsylvania; Penn State College of Medicine, Hershey, Pennsylvania; U.S. Food and Drug Administration, Laurel, Maryland; Penn State College of Agricultural Sciences, University Park, Pennsylvania

## Abstract

**Background:**

Nontyphoidal *Salmonella* (NTS) is a leading cause of foodborne bacterial infections. Annually, ∼200,000 antimicrobial-resistant (AMR) NTS illnesses occur in the United States. NTS infections resistant to ceftriaxone and ciprofloxacin, drugs used for the treatment of severe infections, are a growing concern.

**Methods:**

We identified patient NTS isolates submitted to the Pennsylvania Bureau of Laboratories by two hospitals from the same health system during 2018–2020. All were tested by broth microdilution for susceptibility to antibiotics tracked by the National Antimicrobial Resistance Monitoring System and analyzed by whole-genome sequencing. We identified resistance mechanisms and plasmids with ResFinder and PlasmidFinder, respectively, and compared each isolate in selected serotypes against the PulseNet database to identify genetic relatedness within ≤5 allele differences to non-human sequences uploaded during 2017–2021.

**Results:**

Of 164 human NTS analyzed for susceptibility, 28 (17%) had decreased susceptibility to ciprofloxacin (DSC) [MIC ≥0.12 µg/mL] while 6 (3.7%) were ceftriaxone-resistant. AMR varied by serotype; 8 (50%) *S.* Infantis and 16 (42%) Enteritidis had DSC, representing 86% of all isolates with DSC (Figure). Six ceftriaxone-resistant isolates had genes that confer resistance to third-generation cephalosporins including *bla*_CMY−2_ in 1 Dublin and 2 Typhimurium and *bla*_CTX-M-65_ in 3 Infantis isolates. The 3 Infantis isolates also had a mutation in *gyrA* that results in DSC, plus a known transmissible plasmid IncFIB(pN55391) linked to multiple resistance genes. An Infantis isolate was related to a chicken meat isolate and a Hadar isolate was related to isolates from 3 animals and 3 meat sources. One Dublin isolate was related to 2 isolates from beef in Pennsylvania.

**Figure.**
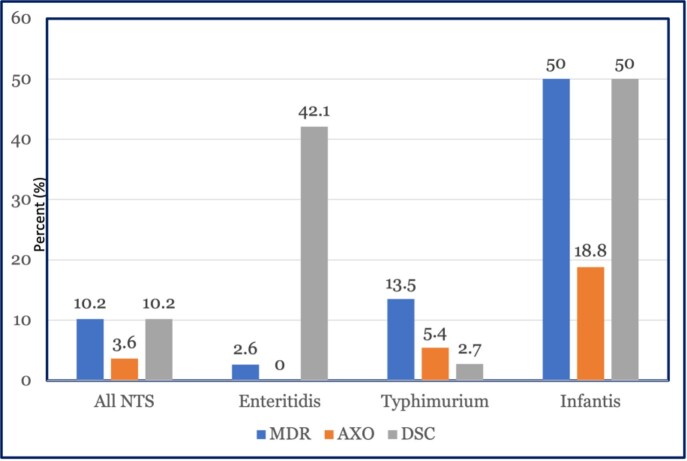
Percent of antimicrobial resistance in nontyphoidal Salmonella (NTS) clinical isolates (n=164) submitted to public health authorities by two hospital systems in Pennsylvania, 2018–2020. Multidrug resistance (MDR) was defined as resistance to ≥3 of the 9 antimicrobial classes tested for NTS clinical isolates. Resistance to ceftriaxone (AXO), a third-generation cephalosporin preferred for treating severe infections in children, was over 5% and 19% in serotypes S. Typhimurium and S. Infantis, respectively. Half (8/16) of Infantis isolates and 42% (16/38) of Enteritidis isolates had decreased susceptibility to ciprofloxacin (DSC).

**Conclusion:**

Ceftriaxone resistance and DSC in NTS from patients in Pennsylvania varied by serotype and some isolates harbored *bla*_CMY−2_ and *bla*_CTX-M-65_ genes. Dissemination of mechanisms that confer resistance to ceftriaxone and DSC is concerning as is the genetic relatedness of isolates from human and animal sources. Our study highlights the need for enhanced One Health AMR monitoring combined with a review of animal production and food processing practices.

**Disclosures:**

**All Authors**: No reported disclosures.

